# Efficacy of Vascular Ligation for the Prevention of Intra- and Postoperative Bleeding in Transoral Robotic Surgery for Oropharyngeal Cancer

**DOI:** 10.3390/cancers17091446

**Published:** 2025-04-25

**Authors:** Tsutomu Ueda, Takayuki Taruya, Minoru Hattori, Nobuyuki Chikuie, Yuki Sato, Takayoshi Hattori, Takao Hamamoto, Takashi Ishino, Sachio Takeno

**Affiliations:** 1 Department of Otorhinolaryngology, Head and Neck Surgery, Graduate School of Biomedical Sciences, Hiroshima University, Hiroshima 734-8551, Japan; ttaruya@hiroshima-u.ac.jp (T.T.); housejak@hiroshima-u.ac.jp (N.C.); sato0123@hiroshima-u.ac.jp (Y.S.); hatt@hiroshima-u.ac.jp (T.H.); takao0320@hiroshima-u.ac.jp (T.H.); tishino@hiroshima-u.ac.jp (T.I.); takeno@hiroshima-u.ac.jp (S.T.); 2 Center for Medical Education Institute of Biomedical & Health Sciences, Hiroshima University, Hiroshima 734-8551, Japan; m-hattori@hiroshima-u.ac.jp

**Keywords:** transoral robotic surgery, oropharyngeal cancer, vascular ligation, neck dissection, intra-operative bleeding, postoperative bleeding

## Abstract

Background: Transoral robotic surgery (TORS) is a minimally invasive procedure that is performed with neck dissection (ND) and postoperative radiotherapy when necessary. This study aimed to review the methods of vascular ligation and ND in cases of TORS for oropharyngeal cancer in Japan. Methods: We enrolled 44 consecutive patients who underwent TORS for laryngopharyngeal cancer between December 2019 and December 2023. Of these, 35 patients who underwent TORS as a first-line treatment for oropharyngeal cancer were included in this study. We retrospectively collected patient data on age, sex, primary tumor location, clinical tumor–node classification, Eastern Cooperative Oncology Group performance status, history of irradiation to the neck, presence of anticoagulants, pathological results, tumor size, total operative duration, console time, length of skin incision operative result, estimated blood loss, late cervical lymph node metastasis, perioperative complications, postoperative hospital stay, postoperative bleeding, period until oral intake after surgery, and swallowing function. Intra- and postoperative outcomes of TORS, TORS + ND (IIa) + vascular ligation, and TORS + ND (II–IV) + vascular ligation. Results: Significant differences were found in operative duration, blood loss during ND, and skin incision length between TORS + ND (IIa) + vascular ligation and TORS + ND (II–IV) + vascular ligation. Console time and blood loss did not significantly differ between the two groups. Each group contained one case of postoperative bleeding. Conclusions: Safe and minimally invasive treatments can be established if vascular ligation and ND are implemented based on appropriate case selection.

## Simple Summary

Transoral robotic surgery is a minimally invasive procedure, but the target patient, oropharyngeal cancer, has often cervical lymph node metastases and also requires level II–IV neck dissection. The risk of intra- and postoperative bleeding is high, and vascular ligation is often required. We evaluated the methods of vascular ligation and neck dissection in transoral robotic surgery for oropharyngeal cancer in Japan. The results show that a safe and minimally invasive treatment can be established if vascular ligation and neck dissection are performed based on appropriate case selection.

## 1. Introduction

Chemoradiotherapy (CRT) is a standard treatment for oropharyngeal cancer. Although CRT is mainly used in advanced oropharyngeal cancer for organ preservation, radiotherapy may be avoided, especially in early-stage cancers, owing to the occurrence of late, adverse events and the development of metachronous double cancers. Transoral robotic surgery (TORS) was first described by Weinstein et al. in 2005 [1] and has become a popular, minimally invasive procedure, particularly for the treatment of patients with early-stage oropharyngeal cancer. In Japan, the use of TORS for laryngeal and pharyngeal cancers has spread rapidly throughout the country since it received coverage under public insurance in April 2022 [2,3,4]. Several reports have shown that TORS alone, owing to its high surgical precision [5,6], is superior to conventional surgery and CRT in terms of postoperative function and quality of life [7,8].

However, challenges are associated with TORS, including intra- and postoperative bleeding and the management of cervical lymph node metastases. Oropharyngeal cancers are surrounded by many blood vessels, and the surgical space is limited. Because bleeding is difficult to manage during and after TORS, preoperative ligation of the lingual, facial, and external carotid arteries is recommended [9,10]. Worldwide, prophylactic level II–IV neck dissection (ND) is often performed in N0 patients undergoing vascular ligation [10].

In Japan, the current indications for TORS for oropharyngeal carcinoma are mainly T1 and T2 cases, regardless of the presence or absence of cervical lymph node metastasis, which do not result in postoperative dysfunction. CRT is recommended for patients with extranodal extension (ENE). Vascular ligation before TORS is recommended for oropharyngeal carcinoma of the lateral or anterior wall, where the risk of intra- and postoperative bleeding is high. This study aimed to review the methods of vascular ligation and ND in cases of TORS for oropharyngeal cancer performed in our department according to an original Japanese concept.

## 2. Patients and Methods

### 2.1. Study Design and Population

A total of 44 consecutive patients who underwent TORS for laryngopharyngeal cancer between December 2019 and December 2023 were retrospectively enrolled in this study. Of these, 35 patients who underwent TORS as a first-line treatment for oropharyngeal cancer were included. None of the patients had any history of oropharyngeal carcinoma treatment.

Patient data on age, sex, primary tumor location, clinical tumor–node classification, Eastern Cooperative Oncology Group performance status, history of irradiation to the neck, presence of anticoagulants, pathological results, tumor size, total operative duration, console time, length of skin incision, operative result, estimated blood loss, late cervical lymph node metastasis, perioperative complications, postoperative hospital stay, postoperative bleeding, period until oral intake after surgery, and swallowing function were retrospectively collected from electronic medical records.

Postoperative bleeding was classified using a scale developed by Pollei et al. [11]. Minor bleeding was defined as the resolution of bleeding without the need for operative intervention, physician evaluation, emergency department visit, or hospital admission. Intermediate bleeding was defined as bleeding that required an operation for control or evaluation. Major bleeding was defined as bleeding that required interventional radiologic embolization or transcervical vessel ligation using the ND incision. Severe bleeding was defined as bleeding that required a blood transfusion or urgent performance of an awake tracheostomy for airway compromise.

Swallowing function was evaluated using the Functional Outcome Swallowing Scale (FOSS) preoperatively and two months postoperatively. The FOSS categorizes swallowing function into six stages: stage 0, normal function and asymptomatic; stage 1, normal function with episodic or daily symptoms of dysphagia; stage 2, compensated abnormal function manifested by significant dietary modifications or prolonged mealtime without weight loss or aspiration; stage 3, decompensated abnormal function with loss of ≤10% of the patient’s body weight over six months owing to dysphagia or daily cough, gagging, or aspiration during meals; stage 4, severely decompensated abnormal function with loss of >10% of the patient’s body weight over six months owing to dysphagia or severe aspiration with bronchopulmonary complications, with nonoral feeding recommended for most nutrition; and stage 5, nonoral feeding for all nutritional needs.

### 2.2. Surgical Procedure

All TORS procedures were performed using the da Vinci Xi Surgical System (Intuitive Surgical Inc., Sunnyvale, CA, USA), with either the Feyh–Kastenbauer retractor modified by Weinstein–O’Malley (Olympus, Tokyo, Japan), Crowe–Davis retractor (Nagashima Medical Instruments, Tokyo, Japan), or Satou’s Curved Laryngo-Pharyngoscope with a mouth gag frame (Type-S2) (Nagashima Medical Instruments). Resection was performed using monopolar curved scissors or a permanent cautery spatula. Hemostasis was achieved using Maryland bipolar forceps, and titanium surgical clips were used for all exposed vessels.

**Figure 1:**
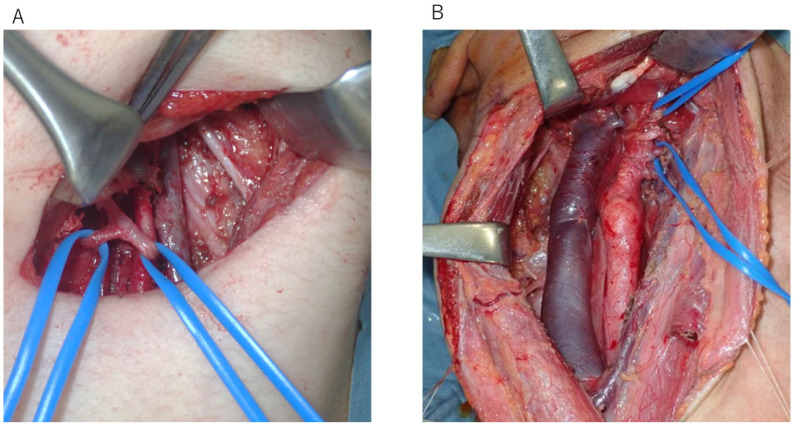
(**A**) In cN0 cases, level IIa neck dissection and ligation of the lingual and facial arteries were performed for anterior and lateral wall cancers with high bleeding potential. (**B**) In cN+ cases, level II–IV neck dissection and ligation of the lingual and facial arteries were performed.

In cN0 cases, level IIa ND and ligation of the lingual and facial arteries were performed for anterior and lateral wall cancers with high bleeding potential (Figure 1A).

CRT was recommended instead of TORS for cN+ cases with high probabilities of ENE. Furthermore, cN+ cases with possible ENE underwent ND according to the previous schedule, and TORS was performed after pathology results confirmed that ENE was negative. Level II–IV ND and TORS were performed on the same day in patients with low preoperative likelihoods of ENE. The lingual and facial arteries were ligated in all cases (Figure 1B).

### 2.3. Ethical Considerations

This study was approved by the review board of our institution (approval number: E-2039) and was conducted in accordance with the principles of the Declaration of Helsinki. The study protocol was posted at our institution, and all patients were given the choice to opt out of this study.

### 2.4. Statistical Analysis

All statistical analyses were performed using IBM SPSS Statistics for Windows version 27 (IBM Corp., Armonk, NY, USA). Continuous variables are presented as medians (ranges). Categorical variables are presented as numbers (percentages). In this study, we aimed to address two distinct clinical questions: (1) how TORS alone compares with TORS accompanied by neck dissection and vascular ligation, and (2) how the extent of neck dissection (level IIa vs. level II–IV) impacts surgical outcomes. For these focused and clinically relevant comparisons, we used the Mann–Whitney U test and Fisher’s exact test, which are appropriate for analyzing two independent groups. Statistical significance was set at *p* < 0.05.

## 3. Results

### 3.1. Baseline Patient Characteristics

Patient characteristics are listed in Table 1. The median age at the time of treatment was 71.0 years (range, 30–81 years), and 82.9% of the patients were male. The cT classification was as follows: cTis, 7 patients (20.0%); cT1, 11 patients (31.4%); cT2, 16 patients (45.7%); and cT4a, 1 patient (2.9%).

The primary lesions were located in the lateral wall in 17 patients (48.6%), anterior wall in 10 (28.6%), superior wall in 5 (14.3%), and posterior wall in 3 (8.6%). The pathology results showed 25 cases of squamous cell carcinoma (SCC), 7 cases of SCC in situ, and 1 case each of basal cell carcinoma, clear cell carcinoma, and mucoepidermoid carcinoma. Additionally, 13 patients (37.1%) were p16-positive. The N classification was cN0 in 28 patients (80.0%), cN1 in 5 (14.3%), and cN2 in 2 (5.7%). Furthermore, seven patients (20.0%) had previously received radiation to the neck, and seven (20.0%) received perioperative anticoagulation. None of the patients had any relevant treatment history. Of the 15 patients (42.9%) who underwent ND, 8 (22.9%) underwent level II, and 7 (20%) underwent level II–IV dissection. All seven cTis cases were treated using TORS only. Patients who underwent TORS and level IIa ND with vascular ligation (TORS + ND [IIa] + vascular ligation) had primary sites at the palatine tonsil and base of the tongue at risk of bleeding with cN0. Patients with cN+ underwent TORS and level II–IV ND with vascular ligation (TORS + ND [II–IV] + vascular ligation). The cT4a case had a primary site at the palatine tonsil with mild invasion of the palatoglossus muscle. Our multidisciplinary team determined that TORS without reconstruction was an option; therefore, TORS was performed despite the presence of a T4a stage. All patients had preoperative FOSS stages of 0.

### 3.2. Assessment of Intra- and Postoperative Outcomes

The assessment of intra- and postoperative outcomes for TORS alone and TORS with level IIa or level II–IV ND with vascular ligation (TORS + ND + vascular ligation) are summarized in Table 2. The median total operative duration was significantly shorter for TORS than that for TORS + ND + vascular ligation (77.5 min [range, 42.0–132.0 min] and 202.0 min [range, 122.0–398.0 min], respectively; *p* < 0.001). The median console time was significantly shorter for TORS than that for TORS + ND + vascular ligation (58.0 min [range, 40.0–103.0 min] and 91.0 min [range, 68.0–163.0 min], respectively; *p* < 0.001). The median tumor size was significantly smaller for TORS than for TORS + ND + vascular ligation (16.5 mm [range, 5.0–39.0 mm] and 26.0 mm [range, 18.0–40.0 mm], respectively; *p* = 0.012). The median total blood loss was significantly lower for TORS than for TORS + ND + vascular ligation (2.5 mL [range, 0.0–60.0 mL] and 30.0 mL [range, 0.0–170.0 mL], respectively). Two cases of late lymph node metastasis developed in each group, with no significant difference in incidence. The overall incidence of postoperative bleeding was 8.6% (3/35). No significant difference was observed in the incidence of postoperative bleeding between patients who underwent TORS (1/20, 5.0%) and those who underwent TORS + ND + vascular ligation (2/15, 13.3%; *p* = 0.565). The median postoperative hospital stay was significantly shorter for TORS than that for TORS + ND + vascular ligation (6 days [range, 3–14 days] and 10 days [range, 6–32 days], respectively). The median period until oral intake after surgery and the FOSS score two months after surgery did not significantly differ between the two groups.

The assessment of intra- and postoperative outcomes in TORS + ND (IIa) + vascular ligation and TORS + ND (II–IV) + vascular ligation are summarized in Table 3. The median total operative duration was significantly shorter in TORS + ND (IIa) + vascular ligation than that in TORS + ND (II–IV) + vascular ligation (172.5 min [range, 122.0–261.0 min] and 307.0 min [range, 202.0–398.0 min], respectively; *p* = 0.003). The median ND duration was significantly shorter in TORS + ND (IIa) + vascular ligation than that in TORS + ND (II–IV) + vascular ligation (84.0 min [range, 50.0–134.0] and 199.0 min [range, 112.0–269.0 min]; *p* = 0.003). The median tumor size, console time, and blood loss during the console did not significantly differ between TORS + ND (IIa) + vascular ligation and TORS + ND (II–IV) + vascular ligation. The median total blood loss was significantly lower in TORS + ND (IIa) + vascular ligation than that in TORS + ND (II–IV) + vascular ligation (15.0 mL [range, 0.0–48.0 mL] and 43.0 mL [range, 22.0–1708.0 mL], respectively). The median total blood loss was significantly higher in TORS + ND (IIa) + vascular ligation than that in TORS + ND (II–IV) + vascular ligation (33.0 mL [range, 10.0–160.0 mL] and 7.5 mL [range, 0.0–24.0 mL], respectively). The median length of skin incision was significantly shorter in TORS + ND (IIa) + vascular ligation than that in TORS + ND (II–IV) + vascular ligation (4.0 cm [range, 3.0–5.0 cm] and 12.0 cm [range, 10.0–17.0 cm], respectively). Of the eight patients who underwent TORS + ND (IIa) + vascular ligation at N0, three patients had pathologically positive lymph nodes, but no additional treatment was given. One case of late lymph node metastasis developed in each group, with no significant difference in incidence. Late lymph node metastasis in cases with TORS + ND (IIa) + vascular ligation contained pathologically positive lymph nodes, and the site of late lymph node metastasis was IIb. No significant difference was identified in the incidence of postoperative bleeding between patients who had TORS + ND (IIa) + vascular ligation (1/8, 12.5%) and those who had TORS + ND (II–IV) + vascular ligation (1/7, 14.3%). The median postoperative hospital stay, period until oral intake after surgery, and FOSS score at two months after surgery were not significantly different between the two groups.

### 3.3. Postoperative Bleeding After TORS

All patients who experienced postoperative bleeding after TORS are listed in Table 4. The bleeding severity classification was minor in two cases and intermediate in one, and no prior cases of radiation exposure were reported. Perioperative anticoagulation was administered to one of the three cases. One case did not involve vascular ligation, one case involved ND (IIa) + vascular ligation, and one case involved TORS + ND (II–IV) + vascular ligation. In all three cases, the intra-operative blood loss was <15 mL. The time to postoperative bleeding ranged from 10 to 14 days. All patients experienced bleeding after hospital discharge.

### 3.4. Late Cervical Lymph Node Metastasis After TORS

All cases of late cervical lymph node metastasis after TORS are shown in Table 5. Two cases did not involve vascular ligation, one case involved ND (IIa) + vascular ligation, and one case involved TORS + ND (II–IV) + vascular ligation. The primary lesion was in the palatine tonsil in all cases. The time to late cervical lymph node metastasis ranged from 3 to 18 months. In the cases of ND, the late cervical lymph node site was located outside the dissection.

## 4. Discussion

TORS is generally indicated for cTis, 1, and 2; ND, including vascular ligation, is not performed if the risk of bleeding is considered low in cN0. In cN0 cases, level IIa ND and ligation of the lingual and facial arteries were performed for anterior and lateral wall cancers with high bleeding potential. Level II–IV ND was not performed in N0 cases. In cN+ cases, level II–IV ND and ligation of the lingual and facial arteries were performed. Furthermore, postoperative CRT and radiotherapy were not performed unless the patient was at a high risk postoperatively. In this study, postoperative CRT was performed in only one patient who had close margins.

Cases in which ND was not required had a significantly smaller tumor size, shorter operative time, shorter postoperative hospital stay, and significantly less blood loss than cases that required ND. This suggests that appropriate preoperative diagnosis and treatment strategies may benefit patients. Of the patients with cN0 who did not undergo ND, two (10%) had late lymph node metastases, but the disease was controlled by careful follow-up and subsequent ND.

Although we compared TORS alone with TORS + ND + vascular ligation, these groups differed markedly in baseline characteristics—most notably tumor size (median 16.5 mm vs. 26.0 mm, *p* = 0.012) and nodal status—factors that guided the surgical choice. Consequently, this comparison is exploratory and cannot yield definitive causal inference; any outcome differences must therefore be interpreted with caution. Our primary analysis instead examines the methodologically comparable subgroups TORS + ND (IIa) versus TORS + ND (II–IV), in which vascular ligation was uniformly applied, and the case-selection criteria were consistent. This targeted comparison provides more clinically meaningful insights into the potential benefits of limiting the extent of neck dissection in appropriately selected patients.

In cN0 cases, IIa + ligation has the advantage of significantly shorter skin scars and significantly shorter time for neck dissection than TORS + ND (II–IV) + vascular ligation. Considering that the rates of postoperative hemorrhage and posterior lymph node metastases were similar in each group, including TORS-only cases, we consider IIa + ligation to be a strategy for the cN0 cases at risk of bleeding.

Concerning postoperative swallowing function, patients had FOSS stage 0 at two months postoperatively, except for one patient who had FOSS stage 1. Outside Japan, ND and postoperative radiation are often performed together with TORS. Long-term results of the ORATOR Trial reported better swallowing with radiotherapy (80% CRT) than that with TORS with ND [12]. Furthermore, Campo et al. [13] reported no difference in swallowing between TORS and radiotherapy (80% CRT) in a meta-analysis. However, the former study was influenced by the fact that 71% of the patients in the TORS group received postoperative radiotherapy, and the latter study showed that 54% of the TORS group received postoperative treatment. However, the discussion of the ORATOR Trial described that their post hoc analysis showed no differences in swallowing quality of life on the basis of whether patients received TORS plus ND alone, TORS plus ND and radiotherapy, or TORS plus ND and chemoradiotherapy, suggesting that differences in adjuvant treatment did not affect their primary endpoint and that case selection is important for preserving swallowing functions in TORS [12].

In contrast, Scott et al. [14] reported that TORS (19.6% postoperative CRT) at three-year follow-up had a better objective swallowing function than CRT. Charters et al. [15] reported better swallowing with TORS alone or postoperative radiotherapy (cervical only) than that with TORS plus postoperative radiotherapy (including primary) or CRT.

This exploratory study suggested that the treatment strategy for selecting patients for TORS is justified if postoperative radiotherapy or CRT can be omitted or limited to the neck. Costantino et al. [16] reported that neoadjuvant chemotherapy and TORS can achieve excellent tumor control and survival in locoregionally advanced oropharyngeal cancers. Therefore, neoadjuvant chemotherapy may reduce the need for adjuvant treatment [17,18,19,20].

Regarding intra-operative bleeding, no obvious differences in console bleeding with or without vascular ligation were observed; however, considering that cases without vascular ligation were smaller in size and less likely to bleed and that the amount of blood loss was equivalent to that in those cases, vascular ligation was considered effective.

Overall, 30- and 90-day perioperative mortality rates for TORS for T1 and T2 oropharyngeal SCC have been reported to be 0.6% (23/4, 127) and 0.9% (38/4, 127), respectively [21]. Most perioperative deaths have been attributed to postoperative bleeding [22,23]; hence, reliable control of bleeding is necessary. The incidence of postoperative bleeding has been reported to range from 1.5 to 16% [9,10,22,23,24,25]. Mackie et al. [26] compared total external carotid artery resection with selective ligation in patients undergoing transoral resection for oropharyngeal squamous cell carcinoma of the base of tongue or palatine tonsils. The results showed that selective ligation of external carotid vessels versus total ligation of the external carotid artery shows similar rates of oropharyngeal bleeding. We agree that ligation is necessary for invasive cancer. All cases in which we ligated for invasive cancer were selective ligations and selective ligation will continue in the future. Only three cases (8.6%) with minor and intermediate bleeding were found in our study. Considering that postoperative bleeding was observed in cases of no ND, level IIa ND and vascular ligation, and level II–IV ND and vascular ligation, and that the time of bleeding was after discharge in all cases, careful postoperative wound follow-up and frequent post-discharge visits are necessary. The usefulness of vascular ligation in postoperative bleeding has also been reported [27,28,29,30]; however, no significant differences were found in the present study. In a previous study, no complications from vascular ligation were noted, and severe bleeding only occurred in 1 of the 174 (0.6%) patients who underwent prophylactic ligation [9]. No complications from vascular ligation were observed, and no serious bleeding occurred in the present study. Therefore, future studies should consider vascular ligation.

This study had some limitations. As this study was conducted at a single center, the sample size was small, particularly in the subgroup analysis comparing TORS + ND (IIa) and TORS + ND (II–IV). Therefore, our findings should be interpreted as preliminary. Further investigation in larger, multicenter studies is necessary to validate the impact of ND extent on outcomes. However, given the rarity of TORS with ND procedures in Japan, we believe our findings offer clinically relevant insights and may serve as a valuable basis for future hypothesis-driven research.

## 5. Conclusions

This is the first report to examine vascular ligation and ND selection methods in TORS. This study suggests that a safe and minimally invasive treatment can be established if vascular ligation and ND are performed based on appropriate case selection.

## Figures and Tables

**Table 1:** Patient characteristics.

Characteristics	Group	All Patients (N = 35)	TORS (N = 20)	TORS + ND (IIa) + Vascular Ligation (N = 8)	TORS + ND (II–IV) + Vascular Ligation (N = 7)
Age, year median		71.00 [30.00, 81.00]	71.00 [59.00, 80.00]	71.50 [30.00, 77.00]	64.00 [37.00, 81.00]
Sex, No. (%)	male	29 (82.9)	17 (85.0)	6 (75.0)	6 (85.7)
	Female	6 (17.1)	3 (15.0)	2 (25.0)	1 (14.3)
PS, No. (%)	0	34 (97.1)	19 (95.0)	8 (100.0)	7 (100.0)
	1	1 (2.9)	1 (5.0)	0 (0.0)	0 (0.0)
Perioperative anticoagulation, No. (%)	Yes	7 (20.0)	4 (20.0)	2 (25.0)	1 (14.3)
	No	28 (80.0)	16 (80.0)	6 (75.0)	6 (85.7)
History of head and neck cancer	Yes	9 (25.7)	7 (35.0)	1 (12.5)	1 (14.3)
	No	26 (74.3)	13 (65.0)	7 (87.5)	6 (85.7)
Prior radiation, No. (%)	Yes	7 (20.0)	5 (25.0)	1 (12.5)	1 (14.3)
	No	28 (80.0)	15 (75.0)	7 (87.5)	6 (85.7)
Primary site, No. (%)	Soft palate	5 (14.3)	4 (20.0)	0 (0.0)	1 (14.3)
	Palatine tonsil	17 (48.6)	9 (45.0)	3 (37.5)	5 (71.4)
	Base of tongue	10 (28.6)	4 (20.0)	5 (62.5)	1 (14.3)
	Posterior wall	3 (8.6)	3 (15.0)	0 (0.0)	0 (0.0)
Pathological diagnosis (%)	BCC	1 (2.9)	0 (0.0)	0 (0.0)	1 (14.3)
	clear cell ca	1 (2.9)	0 (0.0)	1 (12.5)	0 (0.0)
	mucoeidermoid carcinoma	1(2.9)	1 (5.0)	0 (0.0)	0 (0.0)
	SCC	25 (71.4)	12 (60.0)	7 (87.5)	6 (85.7)
	SCCinsitu	7 (20.0)	7 (35.0)	0 (0.0)	0 (0.0)
p16status, No. (%)	Positive	13 (37.1)	6 (30.0)	2 (25.0)	5 (71.4)
	Negative	22 (62.9)	14 (70.0)	6 (75.0)	2 (28.6)
cT (%), No. (%)	is	7 (20.0)	7 (35.0)	0 (0.0)	0 (0.0)
	1	11 (31.4)	7 (35.0)	2 (25.0)	2 (28.6)
	2	16 (45.7)	6 (30.0)	5 (62.5)	5 (71.4)
	4a	1 (2.9)	0 (0.0)	1 (12.5)	0 (0.0)
cN (%), No. (%)	0	28 (80.0)	20 (100.0)	8 (100.0)	0 (0.0)
	1	5 (14.3)	0 (0.0)	0 (0.0)	5 (71.4)
	2	2 (5.7)	0 (0.0)	0 (0.0)	2 (28.6)

**Table 2:** Assessment of intra- and postoperative outcomes (TORS vs. TORS + ND).

Factor	Group	TORS (N = 20)	TORS + ND + Vascular Ligation (N = 15)	*p* Value
Total operation time, min		77.5 [42.0, 132.0]	202.0 [122.0, 398.0]	<0.001
Console time, min		58.0 [40.0, 103.0]	91.0 [68.0, 163.0]	<0.001
Total amount of blood loss, mL		2.50 [0.0, 60.0]	30.0 [0.0, 170.0]	0.001
blood loss during console		2.50 [0.0, 60.0]	10.0 [0.0, 34.0]	0.221
blood loss during ND		NA	13.0 [0.0, 160.0]	-
tumor size, mm		16.50 [5.0, 39.0]	26.0 [18.0, 40.0]	0.012
Length of skin incision, cm		NA	5.0 [3.00, 17.00]	-
late cervical lymph node metastasis (%)	Yes	2 (10.0)	2 (13.3)	1
	No	18 (90.0)	13 (86.7)	
surgical margin (%)	Yes	0 (0.0)	1 (6.7)	0.429
	No	20 (100.0)	14 (93.3)	
pN (%)	0	19 (100.0)	5 (33.3)	<0.001
	1	0 (0.0)	5 (33.3)	
	2	0 (0.0)	5 (33.3)	
pT (%)	is	7 (35.0)	0 (0.0)	<0.001
	1	7 (35.0)	0 (0.0)	
	2	6 (30.0)	13 (86.7)	
	4	0 (0.0)	2 (13.3)	
SICU stay (%)	Yes	1 (5.0)	1 (6.7)	1
	No	19 (95.0)	14 (93.3)	
perioperative complications (%)	Yes	4 (20.0)	5 (33.3)	0.451
	No	16 (80.0)	10 (66.7)	
postoperative bleeding (%)	Yes	1 (5.0)	2 (13.3)	0.565
	No	19 (95.0)	13 (86.7)	
postoperative hospital stay, day		6.0 [3.0, 14.0]	10.0 [6.0, 32.0]	0.015
period until peroral intake after surgery, day		1.0 [1.0, 11.0]	2.0 [1.0, 26.0]	0.13
FOSS at 2 months after surgery (%)	0	18 (100.0)	13 (92.9)	0.438
	1	0 (0.0)	1 (7.1)	

**Table 3:** Assessment of intra- and postoperative outcomes (TORS + ND [IIa] + vascular ligation) vs. (TORS + ND [II–IV] + vascular ligation).

Factor	Group	TORS + ND (IIa) + Vascular Ligation (N = 8)	TORS + ND (II–IV) + Vascular Ligation (N = 7)	*p* Value
Operation time, min		172.5 [122.0, 261.0]	307.0 [202.0, 398.0]	0.003
Console time, min		88.5 [68.0, 127.0]	91.00 [78.0, 163.0]	0.385
ND time, min		84.0 [50.0, 134.0]	199.0 [112.0, 269.0]	0.003
Total amount of blood loss, mL		15.0 [0.0, 48.0]	43.0 [22.0, 170.0]	0.032
blood loss during console, mL		10.0 [0.0, 34.0]	10.0 [0.0, 20.0]	0.677
blood loss during ND, mL		7.5 [0.0, 24.0]	33.0 [10.0, 160.0]	0.02
tumor size, mm		25.0 [18.0, 40.0]	26.0 [21.0, 37.0]	0.816
Length of skin incision, cm		4.0 [3.0, 5.0]	12.0 [10.0, 17.0]	0.001
late cervical lymph node metastasis (%)	Yes	1 (12.5)	1 (14.3)	1
	No	7 (87.5)	6 (85.7)	
surgical margin (%)	Yes	0 (0.0)	1 (14.3)	0.467
	No	8 (100.0)	6 (85.7)	
pN (%)	0	5 (62.5)	0 (0.0)	0.068
	1	2 (25.0)	3 (42.9)	
	2	1 (12.5)	4 (57.1)	
pT (%)	is	0 (0.0)	0 (0.0)	1
	1	0 (0.0)	0 (0.0)	
	2	7 (87.5)	6 (85.7)	
	4	1 (12.5)	1 (14.3)	
SICU stay (%)	Yes	0 (0.0)	1 (14.3)	0.467
	No	8 (100.0)	6 (85.7)	
perioperative complications (%)	Yes	2 (25.0)	3 (42.9)	0.608
	No	6 (75.0)	4 (57.1)	
postoperative bleeding (%)	Yes	1 (12.5)	1 (14.3)	1
	No	7 (87.5)	6 (85.7)	
postoperative hospital stay, day		10.5 [6.0, 18.0]	10.0 [7.0, 32.0]	0.954
period until peroral intake after TORS, day		2.0 [1.0, 11.0]	2.0 [1.0, 26.0]	0.507
FOSS at 2 months, after TORS (%)	0	7 (87.5)	6 (100.0)	1
	1	1 (12.5)	0 (0.0)	

**Table 4:** Details of patients who experienced postoperative bleeding after TORS.

Sex	Age	Primary Site	Bleeding Severity	Postoperative Day of Bleeding	Postoperative Hospital Stay	Prior Radiation	Perioperative Anticoagulation	Blood Loss During Console	Blood Loss During ND	ND + Vascular Ligation	pT	pN	p16 Status	Surgical Margin
Male	80	Palatine tonsil	minor	10	9	-	-	2	NA	-	1	0	positive	negative
Male	63	Base of tongue	intermediate	14	6	-	-	0	13	ND (IIa) + vascular ligation	2	0	positive	negative
Male	73	Base of tongue	minor	14	10	-	+	15	10	ND (II–IV) + vascular ligation	2	2	positive	negative

**Table 5:** Details of patients who experienced late cervical lymph node metastasis after TORS.

Sex	Age	Primary Site	The Time to Late Cervical Lymph Node Metastasis (Month)	The Late Cervical Lymph Node Site	ND + Vascular Ligation	Pathological Diagnosis	pT	pN	p16 Status	Surgical Margin
Male	71	Palatine tonsil	3	IIa	-	SCC	2	0	positive	negative
Male	80	Palatine tonsil	15	IIa, III	-	SCC	1	0	positive	negative
Male	77	Palatine tonsil	6	IIb	ND (IIa) + vascular ligation	SCC	4a	2	negative	negative
Male	64	Palatine tonsil	18	V, RLN	ND (II–IV) + vascular ligation	SCC	2	1	positive	negative
RLN: Rouviere lymph node										

## Data Availability

The datasets that support the findings of our study are available from the authors on reasonable request.

## Abbreviations

The following abbreviations are used in this manuscript:

TORS	Transoral robotic surgery
ND	Neck dissection
CRT	Chemoradiotherapy
ENE	Extranodal extension
FOSS	Functional Outcome Swallowing Scale
SCC	Squamous cell carcinoma
RLN	Rouviere lymph node
